# Supplementary material for: Effect of a Polyglycolic Acid Mesh Sheet (Neoveil™) in Thyroid Cancer Surgery: A Prospective Randomized Controlled Trial
Source: Cancers (Basel). 2022 Aug 12;14(16):3901. doi: 10.3390/cancers14163901 (PMC9406169; doi:10.3390/cancers14163901)
Supplement: Supplementary file 1 [file cancers-14-03901-s001.zip › cancers-1812767-supplementary.pdf]

Table S1. Postoperative outcome in central lymph node dissection.

Variables	Control (n = 157)	Treatment (n = 154)	*p* value
Drain amount (mL, mean ± sd, 95% CI)			
Postoperative day 1	88.3 ± 42.5 (81.7 - 95.1)	78.3 ± 40.8 (71.7 - 84.4)	0.036
Postoperative day 2	71.1 ± 36.0 (65.8 - 76.4)	59.5 ± 33.8 (54.0 - 65.1)	0.004
Total	159.4 ± 68.1 (149.4 - 170.1)	137.9 ± 66.1 (127.1 - 148.1)	0.005
Triglyceride (mg/dL, mean ± sd, 95% CI)			
Postoperative day 1	93.2 ± 61.0 (84.0 - 103.1)	82.6 ± 59.4 (73.7 - 92.5)	0.121
Postoperative day 2	67.5 ± 99.5 (54.0 - 84.0)	54.2 ± 81.6 (44.5 - 69.3)	0.198

Table S2. Postoperative outcome in central lymph node dissection with lateral lymph node dissection.

Variables	Control (n = 7)	Treatment (n = 7)	*p* value
Drain amount (mL, mean ± sd, 95% CI)			
Postoperative day 1	133.6 ± 48.0 (106.4 - 174.2)	158.3 ± 54.8 (120.5 - 192.6)	0.387
Postoperative day 2	97.4 ± 69.3 (57.4 - 149.6)	90.9 ± 46.6 (63.3 - 127.0)	0.838
Total	231.0 ± 113.1 (169.3 - 322.7)	249.1 ± 94.7 (187.7 - 317.0)	0.750
Triglyceride (mg/dL, mean ± sd, 95% CI)			
Postoperative day 1	67.9 ± 25.7 (51.6 - 85.4)	54.7 ± 30.7 (35.6 - 78.0)	0.403
Postoperative day 2	70.3 ± 97.5 (28.3 - 146.7)	40.7 ± 50.3 (14.7 - 79.7)	0.489
